# Supplementary material for: New Tetrahydroacridine Hybrids with Dichlorobenzoic Acid Moiety Demonstrating Multifunctional Potential for the Treatment of Alzheimer’s Disease
Source: Int J Mol Sci. 2020 May 26;21(11):3765. doi: 10.3390/ijms21113765 (PMC7312527; doi:10.3390/ijms21113765)
Supplement: Supplementary file 1 [file ijms-21-03765-s001.pdf]

International Journal of
Molecular Sciences

MDPI

Supplementary materials

New tetrahydroacridine hybrids with dichlorobenzoic acid moiety demonstrating multifunctional potential for the treatment of Alzheimer's disease

Kamila Czarnecka[1], Małgorzata Girek[1], Przemysław Wójtowicz[1], Paweł Kręcisz[1], Robert Skibiński[2], Jakub Jończyk[3], Kamil Łątka[3], Marek Bajda[3], Anna Walczak[4], Grzegorz Galita[4], Jacek Kabziński[4], Ireneusz Majsterek[4], Piotr Szymczyk[5], Paweł Szymański[1,*]

1 Department of Pharmaceutical Chemistry, Drug Analyses and Radiopharmacy, Faculty of Pharmacy, Medical University of Lodz, Muszyńskiego 1, 90-151 Lodz, Poland; kamila.czarnecka@umed.lodz.pl (K.C); malgorzata.girek@stud.umed.lodz.pl (M.G.); przemyslaw.wojtowicz@stud.umed.lodz.pl (P.W.) pawel.krecisz@stud.umed.lodz.pl (P.K.); pawel.szymanski@umed.lodz.pl (P.Sz.)
2 Department of Medicinal Chemistry, Faculty of Pharmacy, Medical University of Lublin, Jaczewskiego 4, 20-090 Lublin, Poland; robertskibinski@umlub.pl (R.S)
3 Department of Physicochemical Drug Analysis, Chair of Pharmaceutical Chemistry, Faculty of Pharmacy, Jagiellonian University Medical College, Medyczna 9, 30-688 Krakow, Poland; kamil1.latka@uj.edu.pl (K.L.); jakub.jonczyk@doctoral.uj.edu.pl (J.J.); marek.bajda@uj.edu.pl (M.B.)
4 Department of Clinical Chemistry and Biochemistry, Medical University of Lodz, Narutowicza 60, 90-647 Lodz, Poland; jacek.kabzinski@umed.lodz.pl (J.K.); anna.walczak@umed.lodz.pl (A.W.); grzegorz.galita@umed.lodz.pl (G.G.); ireneusz.majsterek@umed.lodz.pl (I.M.)
5 Department of Pharmaceutical Biotechnology, Faculty of Pharmacy, Medical University of Lodz, Muszyńskiego 1, 90-151 Lodz, Poland; piotr.szymczyk@umed.lodz.pl (P.Sz)

* Correspondence: kamila.czarnecka@umed.lodz.pl; pawel.szymanski@umed.lodz.pl;

Table of Contents

General procedure for the synthesis of 2a–2h 4

3,5-dichloro-N-[2-(1,2,3,4-tetrahydroacridin-9-ylamino)ethyl]benzamide (2a)............................ 4

3,5-dichloro-N-[2-(1,2,3,4-tetrahydroacridin-9-ylamino)propyl]benzamide (2b) 4

3,5-dichloro-N-[2-(1,2,3,4-tetrahydroacridin-9-ylamino)butyl]benzamide (2c)............................ 4

3,5-dichloro-N-[2-(1,2,3,4-tetrahydroacridin-9-ylamino)pentyl]benzamide (2d).......................... 4

3,5-dichloro-N-[2-(1,2,3,4-tetrahydroacridin-9-ylamino) hexyl]benzamide (2e) 5

3,5-dichloro-N-[2-(1,2,3,4-tetrahydroacridin-9-ylamino) heptyl]benzamide (2f) 5

3,5-dichloro-N-[2-(1,2,3,4-tetrahydroacridin-9-ylamino) octyl]benzamide (2g) 5

3,5-dichloro-N-[2-(1,2,3,4-tetrahydroacridin-9-ylamino) nonyl]benzamide (2h).......................... 5

General Procedure for the Synthesis of Compounds 3a–3h.. 6

3,5-dichloro-N-[2-(1,2,3,4-tetrahydroacridin-9-ylamino)ethyl]benzamide hydrochloride (3a) 6

3,5-dichloro-N-[2-(1,2,3,4-tetrahydroacridin-9-ylamino)propyl]benzamide hydrochloride (3b)... 6

3,5-dichloro-N-[2-(1,2,3,4-tetrahydroacridin-9-ylamino)butyl]benzamide hydrochloride (3c) 6

3,5-dichloro-N-[2-(1,2,3,4-tetrahydroacridin-9-ylamino)pentyl]benzamide hydrochloride (3d)... 6

3,5-dichloro-N-[2-(1,2,3,4-tetrahydroacridin-9-ylamino) hexyl]benzamide hydrochloride (3e).... 6

3,5-dichloro-N-[2-(1,2,3,4-tetrahydroacridin-9-ylamino) heptyl]benzamide hydrochloride (3f)... 7

3,5-dichloro-N-[2-(1,2,3,4-tetrahydroacridin-9-ylamino) octyl]benzamide hydrochloride (3g)..... 7

3,5-dichloro-N-[2-(1,2,3,4-tetrahydroacridin-9-ylamino) nonyl]benzamide hydrochloride (3h) ... 7

Spectral Data 7

MS spectra for 3,5-dichloro-N-[2-(1,2,3,4-tetrahydroacridin-9-ylamino)ethyl]benzamide (2a) 7

^{1}HNMR spectra for 3,5-dichloro-N-[2-(1,2,3,4-tetrahydroacridin-9-ylamino)ethyl]benzamide (2a) 8

MS spectra for 3,5-dichloro-N-[2-(1,2,3,4-tetrahydroacridin-9-ylamino)propyl]benzamide (2b).. 9

^{1}HNMR spectra for 3,5-dichloro-N-[2-(1,2,3,4-tetrahydroacridin-9-ylamino)propyl]benzamide (2b) 10

MS spectra for 3,5-dichloro-N-[2-(1,2,3,4-tetrahydroacridin-9-ylamino)butyl]benzamide (2c) .. 11

^{1}HNMR spectra for 3,5-dichloro-N-[2-(1,2,3,4-tetrahydroacridin-9-ylamino)butyl]benzamide (2c) 12

MS spectra for 3,5-dichloro-N-[2-(1,2,3,4-tetrahydroacridin-9-ylamino)pentyl]benzamide (2d) 13

^{1}HNMR spectra for 3,5-dichloro-N-[2-(1,2,3,4-tetrahydroacridin-9-ylamino)pentyl]benzamide (2d) 14

MS spectra for 3,5-dichloro-N-[2-(1,2,3,4-tetrahydroacridin-9-ylamino) hexyl]benzamide (2e). 15

^{1}HNMR spectra for 3,5-dichloro-N-[2-(1,2,3,4-tetrahydroacridin-9-ylamino) hexyl]benzamide (2e) 16

MS spectra for 3,5-dichloro-N-[2-(1,2,3,4-tetrahydroacridin-9-ylamino) heptyl]benzamide (2f) 17

^{1}HNMR spectra for 3,5-dichloro-N-[2-(1,2,3,4-tetrahydroacridin-9-ylamino) heptyl]benzamide (2f) 18

MS spectra for 3,5-dichloro-N-[2-(1,2,3,4-tetrahydroacridin-9-ylamino) octyl]benzamide (2g).. 19

^{1}HNMR spectra for 3,5-dichloro-N-[2-(1,2,3,4-tetrahydroacridin-9-ylamino) octyl]benzamide (2g) 20

MS spectra for 3,5-dichloro-N-[2-(1,2,3,4-tetrahydroacridin-9-ylamino) nonyl]benzamide (2h) 21

^{1}HNMR spectra for 3,5-dichloro-N-[2-(1,2,3,4-tetrahydroacridin-9-ylamino) nonyl]benzamide (2h) 22

Molecular Modeling - figures with binding modes of selected compounds........ 23

General procedure for the synthesis of 2a–2h

An aqueous solution of N-methylomorpholine was slowly added to a mixture of tetrahydrofuran (THF), 2-chloro-4,6-dimethoxy-1,3,5-triazine (CDMT), 3,5-dichlorobenzoic acid. The reaction was carried out for about two hours in an ice bath. Then of diamine dissolved in THF was added to the mixture and the reaction continued at room temperature for 24 hours to yield the corresponding intermediates 2a–2h.

3,5-dichloro-N-[2-(1,2,3,4-tetrahydroacridin-9-ylamino)ethyl]benzamide (2a)

Compound 2a was obtained from 1a and 3,5-dichlorobenzoic acid as standard procedure: 2-chloro-4,6-dimethoxy-1,3,5-triazine (CDMT) (0.36 g, 2.07 mM), 3,5-dichlorobenzoic acid (0.40 g, 2.07 mM) and N-methylomorpholine (0.23 ml, 2.07 mM) were mixed. 1a compound (0.50 g, 2.07 mM) dissolved in THF (3 ml) was added. Compound 2a: yellow oil; 75.0% yield; FTIR (ATR) v (cm^{-1}): 748.9; 1236.7; 1316.8; 1449.0; 1563.8; 2937.8; 3252.9; ^{1}H NMR (600 MHz, Methanol-d4) δ 8.48 (1H, d, J = 8.4 Hz, Ar), 7.87 (1H, t, J = 8.2 Hz, Ar), 7.75 (1H, d, J = 7.6 Hz, Ar), 7.67 (1H, t, J = 1.9 Hz, Ar), 7.61 – 7.65 (3H, m, Ar), 4.22 – 4.25 (2H, m, CH_2), 3.77 – 3.80 (2H, m, CH_2), 2.99 (2H, t, J = 6.2 Hz, CH_2), 2.78 (2H, t, J = 6.1 Hz, CH_2), 1.92 – 2.01 (4H, m, CH_2), (protons of NH groups invisible); MS (ESI) (M+1) m/z: 215.9, 199.1, 172.2, 121.1; MS-HR (ESI): calcd. for $C_{22}H_{21}Cl_2N_3O$: 413.10617, found: 413.10588

3,5-dichloro-N-[2-(1,2,3,4-tetrahydroacridin-9-ylamino)propyl]benzamide (2b)

Compound 2b was obtained from 1b and 3,5-dichlorobenzoic acid as standard procedure: CDMT (0.34 g, 1.96 mM), 3,5-dichlorobenzoic acid (0.37 g, 1.96 mM) and N-methylomorpholine (0.22 ml, 1.96 mM) were mixed. 1b compound (0.50 g, 1.96 mM) dissolved in THF (3 ml) was added. Compound 2b: yellow oil; 70.0% yield; FTIR (ATR) v (cm^{-1}): 764.3; 1236.8; 1315.9; 1448.9; 1562.9; 2938.2; 3253.7; ^{1}H NMR (600 MHz, Methanol-d_4) δ 8.40 (1H, d, J = 8.5 Hz, Ar), 7.84 (1H, t, J = 8.2 Hz, Ar), 7.74 (1H, d, J = 9.3 Hz, Ar), 7.67 (2H, d, J = 1.9 Hz, Ar), 7.65 (1H, t, J = 1.9 Hz, Ar), 7.56 (1H, t, J = 8.4 Hz, Ar), 4.07 (2H, t, J = 6.5 Hz, CH_2), 3.53 – 3.56 (2H, m, CH_2), 3.01 (2H, t, J = 6.0 Hz, CH_2), 2.77 (2H, t, J = 5.8 Hz, CH_2), 2.14 (2H, p, J = 6.5 Hz, CH_2), 1.94 – 2.01 (4H, m, CH_2), (protons of NH groups invisible); MS (ESI)(M+1) m/z: 230.0, 199.1, 172.9, 121.1; MS-HR (ESI): calcd. for $C_{23}H_{23}Cl_2N_3O$: 427.12182, found: 427.12128

3,5-dichloro-N-[2-(1,2,3,4-tetrahydroacridin-9-ylamino)butyl]benzamide (2c)

Compound 2c was obtained from 1c and 3,5-dichlorobenzoic acid as standard procedure: CDMT (0.33 g, 1.86 mM), 3,5-dichlorobenzoic acid (0.35 g, 1.86 mM) and N-methylomorpholine (0.20 ml, 1.86 mM) were mixed. 1c compound (0.50 g, 1.86 mM) dissolved in THF (3 ml) was added. Compound 2c: yellow oil; 68.0% yield; FTIR (ATR) v (cm^{-1}): 759.1; 1236.7; 1330.7; 1447.5; 1560.1; 2872.0; 3229.9; ^{1}H NMR (600 MHz, Methanol-d4) δ 8.41 (1H, d, J = 8.6 Hz, Ar), 7.84 (1H, t, J = 8.1 Hz, Ar), 7.73 (1H, d, J = 7.9 Hz, Ar), 7.68 – 7.70 (2H, m, Ar), 7.65 (1H, t, J = 1.9 Hz, Ar), 7.58 (1H, t, J = 7.8 Hz, Ar), 4.04 (2H, t, J = 7.0 Hz, CH_2), 3.42 (2H, t, J = 6.7 Hz, CH_2), 3.01 (2H, t, J = 5.8 Hz, CH_2), 2.72 (2H, t, J = 5.6 Hz, CH_2), 1.90 – 2.00 (6H, m, CH_2), 1.77 (2H, p, J = 6.8 Hz, CH_2), (protons of NH groups invisible); MS (ESI)(M+1) m/z: 211.1, 199.1, 172.2, 121.1; MS-HR (ESI): calcd. for $C_{24}H_{25}Cl_2N_3O$: 441.13747, found: 441.13704

3,5-dichloro-N-[2-(1,2,3,4-tetrahydroacridin-9-ylamino)pentyl]benzamide (2d)

Compound 2d was obtained from 1d and 3,5-dichlorobenzoic acid as standard procedure and gave compound 2d: CDMT (0.31 g, 1.76 mM), 3,5-dichlorobenzoic acid (0.34 g, 1.76 mM) and N-methylomorpholine (0.19 ml, 1.76 mM) were mixed. 1d compound (0.50 g, 1.76 mM) dissolved in THF (3 ml) was added. Compound 2d: yellow oil; 76.0% yield; FTIR (ATR) v (cm^{-1}): 767.3; 1236.8; 1330.9; 1451.0; 1559.1; 2967.2; ^{1}H NMR (600 MHz, Methanol-d4) δ 8.41 (1H, d, J = 8.7 Hz, Ar), 7.85 (1H, t, J = 8.1 Hz, Ar), 7.73 – 7.76 (3H, m, Ar), 7.66 (1H, t, J = 1.9 Hz, Ar), 7.59 (1H, t, J = 8.4 Hz, Ar), 3.99 (2H, d, J = 7.1 Hz, CH_2), 3.41 (2H, t, J = 6.9 Hz, CH_2), 3.00 – 3.03 (2H, m, CH_2), 2.70 – 2.73 (2H, m, CH_2), 1.88 – 1.97 (6H, m, CH_2), 1.67 – 1.72 (2H, m, CH_2), 1.52 (2H, p, J = 7.7, 7.2 Hz, CH_2), (protons of NH groups invisible); MS (ESI) (M+1) m/z: 211.1, 199.1, 174.9, 121.1; MS-HR (ESI): calcd. for $C_{25}H_{27}Cl_2N_3O$: 455.15312, found: 455.15232

3,5-dichloro-N-[2-(1,2,3,4-tetrahydroacridin-9-ylamino) hexyl]benzamide (2e)

Compound 2e was obtained from 1e and 3,5-dichlorobenzoic acid as standard: CDMT (0.30 g, 1.68 mM), 3,5-dichlorobenzoic acid (0.32 g, 1.68 mM) and N-methylomorpholine (0.18 ml, 1.68 mM) were mixed. 1e compound (0.50 g, 1.68 mM) dissolved in THF (3 ml) was added. Compound 2e: yellow oil; 60.0% yield; FTIR (ATR) ν (cm^{-1}): 767.3; 1236.6; 1331.9; 1452.9; 1559.1; 2947.6; 469,16877; ^{1}H NMR (600 MHz, Methanol-d4) δ 8.40 (1H, d, J = 8.6 Hz, Ar), 7.85 (1H, t, J = 7.7 Hz, Ar), 7.75 – 7.78 (3H, m, Ar), 7.64 (1H, t, J = 1.9 Hz, Ar), 7.59 (1H, t, J = 8.3 Hz, Ar), 3.97 (2H, t, J = 3.6 Hz, CH_2), 3.38 – 3.40 (2H, m, CH_2), 3.03 (2H, t, J = 5.7 Hz, CH_2), 2.73 (2H, t, J = 5.4 Hz, CH_2), 1.96 – 2.01 (4H, m, CH_2), 1.87 (2H, p, J = 7.4 Hz, CH_2), 1.66 (2H, p, J = 7.1 Hz, CH_2), 1.43 – 1.55 (4H, m, CH_2), (protons of NH groups invisible); MS (ESI)(M+1) m/z: 211.1, 199.1, 172.9, 121.1; MS-HR (ESI): calcd. for $C_{26}H_{29}Cl_2N_3O$: 469.16877, found: 469.16750

3,5-dichloro-N-[2-(1,2,3,4-tetrahydroacridin-9-ylamino) heptyl]benzamide (2f)

Compound 2f was obtained from 1f and 3,5-dichlorobenzoic acid as standard procedure and gave compound 2f: CDMT (0.28 g, 1.61 mM), 3,5-dichlorobenzoic acid (0.31 g, 1.61 mM) and N-methylomorpholine (0.18 ml, 1.61 mM) were mixed. 1f compound (0.50 g, 1.61 mM) dissolved in THF (3 ml) was added. Compound 2f: yellow oil; 65.0% yield; FTIR (ATR) ν (cm^{-1}): 752.9; 1241.8; 1330.3; 1457.0; 1563.8; 2930.7; 3274.7; ^{1}H NMR (600 MHz, Methanol-d4) δ 8.39 (1H, d, J = 8.6 Hz, Ar), 7.85 (1H, t, J = 8.1 Hz, Ar), 7.76 – 7.78 (3H, m, Ar), 7.64 (1H, t, J = 1.9 Hz, Ar), 7.59 (1H, t, J = 8.4 Hz, Ar), 3.93 – 3.96 (2H, m, CH_2), 3.35 – 3.38 (2H, m, CH_2), 3.03 (2H, t, J = 5.8 Hz, CH_2), 2.72 (2H, t, J = 5.6 Hz, CH_2), 1.95 – 2.01 (4H, m, CH_2), 1.86 (2H, p, J = 7.6 Hz, CH_2), 1.63 (2H, p, J = 7.2 Hz, CH_2), 1.40 – 1.50 (6H, m, CH_2), (protons of NH groups invisible); MS (ESI) (M+1) m/z: 211.1, 199.1, 172.9, 121.1; MS-HR (ESI): calcd. for $C_{27}H_{31}Cl_2N_3O$: 483.18442, found: 483.18357

3,5-dichloro-N-[2-(1,2,3,4-tetrahydroacridin-9-ylamino) octyl]benzamide (2g)

Compound 2g was obtained from 1g and 3,5-dichlorobenzoic acid as standard procedure and gave compound 2g: 2-chloro-4,6-dimethoxy-1,3,5-triazine (CDMT) (0.27 g, 1.54 mM), 3,5-dichlorobenzoic acid (0.29g, 1.54 mM) and N-methylomorpholine (0.17 ml, 1.54 mM) were mixed. 1g compound (0.50 g, 1.54 mM) dissolved in THF (3 ml) was added. Compound 2g: yellow oil; 60.0% yield; FTIR (ATR) ν (cm^{-1}): 761.9; 1242.5; 1330.4; 1447.7; 1559.6; 2925.5; 3305.0; ^{1}H NMR (600 MHz, Methanol-d4) δ 8.39 (1H, d, J = 8.6 Hz, Ar), 7.85 (1H, t, J = 8.1 Hz, Ar), 7.76 – 7.79 (3H, m, Ar), 7.64 (1H, t, J = 1.9 Hz, Ar), 7.59 (1H, t, J = 8.3 Hz, Ar), 3.94 (2H, t, J = 7.3 Hz, CH_2), 3.37 (2H, t, J = 7.1 Hz, CH_2), 3.03 (2H, t, J = 5.8 Hz, CH_2), 2.72 (2H, t, J = 5.3 Hz, CH_2), 1.96 – 2.01 (4H, m, CH_2), 1.84 (2H, p, J = 7.4 Hz, CH_2), 1.62 (2H, p, J = 7.1 Hz, CH_2), 1.37 – 1.48 (8H, m, CH_2), (protons of NH groups invisible); MS (ESI) (M+1) m/z: 211.1, 199.1, 171.1, 121.1; MS-HR (ESI): calcd. for $C_{28}H_{33}Cl_2N_3O$: 497.20007, found: 497.19927

3,5-dichloro-N-[2-(1,2,3,4-tetrahydroacridin-9-ylamino) nonyl]benzamide (2h)

Compound 2h was obtained from 1h and 3,5-dichlorobenzoic acid as standard procedure and gave compound 2h: CDMT (0.26 g, 1.47 mM), 3,5-dichlorobenzoic acid (0.28g, 1.47 mM) and N-methylomorpholine (0.16 ml, 1.47 mM) were mixed. 1h compound (0.50 g, 1.47 mM) dissolved in THF (3 ml) was added. Compound 2h : yellow oil; 64.0% yield; FTIR (ATR) ν (cm^{-1}): 755.7; 1282.1; 1355.5; 1497.4; 1562.1; 2925.1; 3273.4; ^{1}H NMR (600 MHz, Methanol-d4) δ 8.30 (1H, d, J = 8.6 Hz, Ar), 7.74 – 7.79 (4H, m, Ar), 7.63 (1H, t, J = 1.9 Hz, Ar), 7.52 (1H, t, J = 8.3 Hz, Ar), 3.82 (2H, t, J = 7.2 Hz, CH_2), 3.35 – 3.38 (2H, m, CH_2), 3.03 (2H, d, J = 6.0 Hz, CH_2), 2.74 (2H, d, J = 5.7 Hz, CH_2), 1.95 – 1.99 (4H, m, CH_2), 1.78 (2H, p, J = 7.4 Hz, CH_2), 1.58 – 1.64 (2H, m, CH_2), 1.35 – 1.45 (10H, m, CH_2), (protons of NH groups invisible); MS (ESI) (M+1) m/z: 211.1, 199.1, 172.9; MS-HR (ESI): calcd. for $C_{29}H_{35}Cl_2N_3O$: 511.21572, found: 511.22220

General Procedure for the Synthesis of Compounds 3a–3h

All new compounds were dissolved (2a-2h) in methanol and HCl/ether was added. After 24 h precipitates had formed and were isolated by filtration and dried. In this procedure, the 3a–3h compounds were obtained. Their physical and spectral data are listed below.

3,5-dichloro-N-[2-(1,2,3,4-tetrahydroacridin-9-ylamino)ethyl]benzamide hydrochloride (3a)

Compound 3a: Yield: 40%; white solid; mp 238–240 ºC; FTIR (ATR) ν (cm^{-1}): 759.8; 1246.0; 1317.2; 1456.7; 1562.7; 2939.4; 3204.1; ^{1}H NMR (600 MHz, DMSO-d_6) δ 13.52 (1H, s, Ar), 8.94 (1H, t, J = 5.5 Hz, Ar), 8.47 (1H, d, J = 8.6 Hz, Ar), 7.87 (1H, d, J = 7.2 Hz, Ar), 7.84 (1H, s, Ar), 7.83 (1H, s, Ar), 7.70 (1H, s, Ar), 7.60 (1H, t, J = 8.3 Hz, Ar), 4.09 (2H, q, J = 5.8 Hz, CH_2), 3.64 (2H, q, J = 5.8 Hz, CH_2), 2.96 (2H, t, J = 5.4 Hz, CH_2), 2.69 (2H, d, J = 5.4 Hz, 3H), 2.05 – 1.97 (m, 0H), 1.85 – 1.79 (m, 6H), (protons of NH groups invisible); MS (ESI) m/z: 215.9, 199.1, 172.9; MS-HR (ESI) calcd for $C_{22}H_{21}Cl_2N_3O$: 413.10617 (without HCl); found: 413.10649

3,5-dichloro-N-[2-(1,2,3,4-tetrahydroacridin-9-ylamino)propyl]benzamide hydrochloride (3b)

Compound 3b: Yield: 36%; white solid; mp 141–143 ºC; FTIR (ATR) ν (cm^{-1}): 754.5; 1266.8; 1317.0; 1447.7; 1562.4; 2940.2; 3201.0; ^{1}H NMR (500 MHz, DMSO-d_6) δ 13.48 (s, 7H), 8.80 (t, J = 5.6 Hz, 1H), 8.35 (d, J = 8.7 Hz, 1H), 7.81 – 7.75 (m, 2H), 7.71 – 7.72 (2H, m, Ar), 7.48 (t, J = 8.3 Hz, 1H), 3.91 (2H, q, J = 6.4 Hz, CH_2), 3.29 – 3.32 (2H, m, CH_2), 2.88 – 2.94 (2H, m, CH_2), 2.57 – 2.64 (2H, m, CH_2), 1.96 (2H, p, J = 6.5 Hz, CH_2), 1.70 – 1.80 (4H, m, CH_2).), (protons of NH groups invisible); MS (ESI) m/z: 430.1, 230.0, 199.1, 172.9; MS-HR (ESI): calcd. for $C_{23}H_{23}Cl_2N_3O$: 427.12182 (without HCl); found: 427.12242

3,5-dichloro-N-[2-(1,2,3,4-tetrahydroacridin-9-ylamino)butyl]benzamide hydrochloride (3c)

Compound 3c: Yield: 35%; white solid; mp 114–116 ºC; FTIR (ATR) ν (cm^{-1}): 758.6; 1230.1; 1317.3; 1456.2; 1576.4; 2879.5; 3202.5; ^{1}H NMR (500 MHz, DMSO-d_6) δ 13.35 (1H, s, HCl), 8.65 (1H, t, J = 5.5 Hz, Ar), 8.32 – 8.36 (1H, m, Ar), 7.79 – 7.82 (1H, m, Ar), 7.71 – 7.77 (3H, m, Ar), 7.51 (1H, t, J = 7.6 Hz, Ar), 3.85 (2H, q, J = 6.6 Hz, CH_2), 3.23 (2H, q, J = 6.6, 5.8 Hz, CH_2), 2.87 – 2.93 (2H, m, CH_2), 2.57 – 2.62 (2H, m, CH_2), 1.70 – 1.80 (6H, m, CH_2), 1.56 (2H, p, J = 6.7 Hz, CH_2), (protons of NH groups invisible); MS (ESI) m/z: 444.1, 253.2, 199.1, 172.9; MS-HR (ESI): calcd. for $C_{24}H_{25}Cl_2N_3O$: 441.13747 (without HCl); found: 441.13737

3,5-dichloro-N-[2-(1,2,3,4-tetrahydroacridin-9-ylamino)pentyl]benzamide hydrochloride (3d)

Compound 3d: Yield: 43%; white solid; mp 88–90 ºC; FTIR (ATR) ν (cm^{-1}): 760.6; 1238.8; 1338.9; 1456.2; 1607.9; 2880.4; 3200.3; ^{1}H NMR (500 MHz, DMSO-d_6) δ 13.44 (1H, s, HCl), 8.61 – 8.66 (1H, m, Ar), 8.33 – 8.37 (1H, m, Ar), 7.75 – 7.84 (4H, m, Ar), 7.52 (1H, t, J = 8.3 Hz, Ar), 3.82 (2H, q, J = 6.7 Hz, CH_2), 3.21 (2H, q, J = 6.5 Hz, CH_2), 2.89 – 2.95 (2H, m, CH_2), 2.56 – 2.62 (2H, m, CH_2), 1.68 – 1.79 (6H, m, CH_2), 1.50 (2H, p, J = 7.1 Hz, CH_2), 1.33 (2H, p, J = 7.6, 7.2 Hz, CH_2), (protons of NH groups invisible); MS (ESI) m/z: 456,2, 267.2, 199.1, 1729; MS-HR (ESI): calcd. for $C_{25}H_{27}Cl_2N_3O$: 455.15312 (without HCl); found: 455.15203

3,5-dichloro-N-[2-(1,2,3,4-tetrahydroacridin-9-ylamino) hexyl]benzamide hydrochloride (3e)

Compound 3e: Yield: 45%; white solid; mp 86–87 ºC; FTIR (ATR) ν (cm^{-1}): 762.5; 1275.1; 1313.8; 1457.2; 2873.5; 3206.1; ^{1}H NMR (500 MHz, DMSO-d_6) δ 13.52 (1H, s, HCl), 8.66 (1H, t, J = 5.5 Hz, Ar), 8.33 – 8.37 (1H, m, Ar), 7.79 – 7.83 (3H, m, Ar), 7.74 – 7.76 (1H, m, Ar), 7.51 – 7.55 (1H, m, Ar), 3.81 (2H, q, J = 6.6 Hz, CH_2), 3.19 (2H, q, J = 6.8 Hz, CH_2), 2.91 – 2.96 (2H, m, CH_2), 2.58 – 2.63 (2H, m, CH_2), 1.76 – 1.82 (4H, m, CH_2), 1.69 (2H, p, J = 7.1 Hz, CH_2), 1.47 (2H, p, J = 7.0 Hz, CH_2), 1.25 – 1.36 (4H, m, CH_2), (protons of NH groups invisible); MS (ESI) m/z: 430.9, 211.1, 199.1, 171.1; MS-HR (ESI): calcd. for $C_{26}H_{29}Cl_2N_3O$: 469.16877 (without HCl); found: 469.16943

3,5-dichloro-N-[2-(1,2,3,4-tetrahydroacridin-9-ylamino) heptyl]benzamide hydrochloride (3f)

Compound 3f: Yield: 38%; white solid; mp 82–83 ºC; FTIR (ATR) ν (cm^{-1}): 757.5; 1242.8; 1323.3; 1456.2; 2896.4; 3204.3; ^1H NMR (500 MHz, DMSO-d_6) δ 13.61 (1H, s, HCl), 8.67 (1H, t, J = 5.6 Hz, Ar), 8.35 (1H, d, J = 8.7 Hz, Ar), 7.80 – 7.85 (3H, m, Ar), 7.75 (1H, t, J = 1.8 Hz, Ar), 7.53 (1H, t, J = 8.3 Hz, Ar), 3.80 (2H, q, J = 6.6 Hz, CH_2), 3.18 (2H, q, J = 6.7 Hz, CH_2), 2.92 – 2.97 (2H, m, CH_2), 2.57 – 2.62 (2H, m, CH_2), 1.75 – 1.80 (4H, m, CH_2), 1.71 – 1.64 (2H, m, CH_2), 1.45 (2H, p, J = 6.8 Hz, CH_2), 1.22 – 1.32 (6H, m, CH_2), (protons of NH groups invisible); MS (ESI) m/z: 430.9, 211.1, 199.1, 172.9; MS-HR (ESI): calcd. for $C_{27}H_{31}Cl_2N_3O$: 483.18442 (without HCl); found: 483.18360

3,5-dichloro-N-[2-(1,2,3,4-tetrahydroacridin-9-ylamino) octyl]benzamide hydrochloride (3g)

Compound 3g: Yield: 32%; white solid; mp 71–72 ºC; FTIR (ATR) ν (cm^{-1}): 758.6; 1268.1; 1334.5; 1454.0; 2882.2; 3203.0; ^1H NMR (500 MHz, DMSO-d_6) δ 13.54 (1H, s, HCl), 8.66 (1H, t, J = 5.5 Hz, Ar), 8.35 (1H, d, J = 8.7 Hz, Ar), 7.80 – 7.83 (3H, m, Ar), 7.75 (1H, t, J = 1.9 Hz, Ar), 7.51 – 7.55 (1H, m, Ar), 3.80 (2H, q, J = 6.6 Hz, CH_2), 3.18 (2H, q, J = 6.7 Hz, CH_2), 2.92 – 2.96 (2H, m, CH_2), 2.58 – 2.62 (2H, m, CH_2), 1.77 – 1.80 (4H, m, CH_2), 1.68 (2H, p, J = 7.4 Hz, CH_2), 1.45 (2H, p, J = 6.5, 5.7 Hz, CH_2), 1.21 – 1.32 (8H. m, CH_2), (protons of NH groups invisible); MS (ESI) m/z: 430.9, 211.1, 199.1, 172.9; MS-HR (ESI): calcd. for $C_{28}H_{33}Cl_2N_3O$: 497.20007 (without HCl); found: 497.19920

3,5-dichloro-N-[2-(1,2,3,4-tetrahydroacridin-9-ylamino) nonyl]benzamide hydrochloride (3h)

Compound 3h: Yield: 35%; white solid; mp 62–63 ºC; FTIR (ATR) ν (cm^{-1}): 755.4; 1246.5; 1320.2; 1457.2; 2878.5; 3205.1; ^1H NMR (500 MHz, DMSO-d_6) δ 13.41 (1H, s, HCl), 8.64 (1H, t, J = 5.4 Hz, Ar), 8.32 – 8.36 (1H, m, Ar), 7.78 – 7.84 (3H, m, Ar), 7.75 (1H, t, J = 1.9 Hz, Ar), 7.53 (1H, t, J = 8.3 Hz, Ar), 3.80 (2H, q, J = 6.8 Hz, CH_2), 3.19 (2H, q, J = 6.9, 6.5 Hz, CH_2), 2.91 – 2.96 (2H, m, CH_2), 2.57 – 2.63 (2H, m, CH_2), 1.77 – 1.81 (4H, m, CH_2), 1.67 (2H, p, J = 7.5 Hz, CH_2), 1.44 (2H, p, J = 7.5, 6.9 Hz, CH_2), 1.20 – 1.31 (10H, m, CH_2), (protons of NH groups invisible); MS (ESI) m/z: 430.9, 211.1, 199.1, 172.9; MS-HR (ESI): calcd. for $C_{29}H_{35}Cl_2N_3O$: 511.21572 (without HCl); found: 511.2148

Spectral Data

MS spectra for 3,5-dichloro-N-[2-(1,2,3,4-tetrahydroacridin-9-ylamino)ethyl]benzamide (2a)

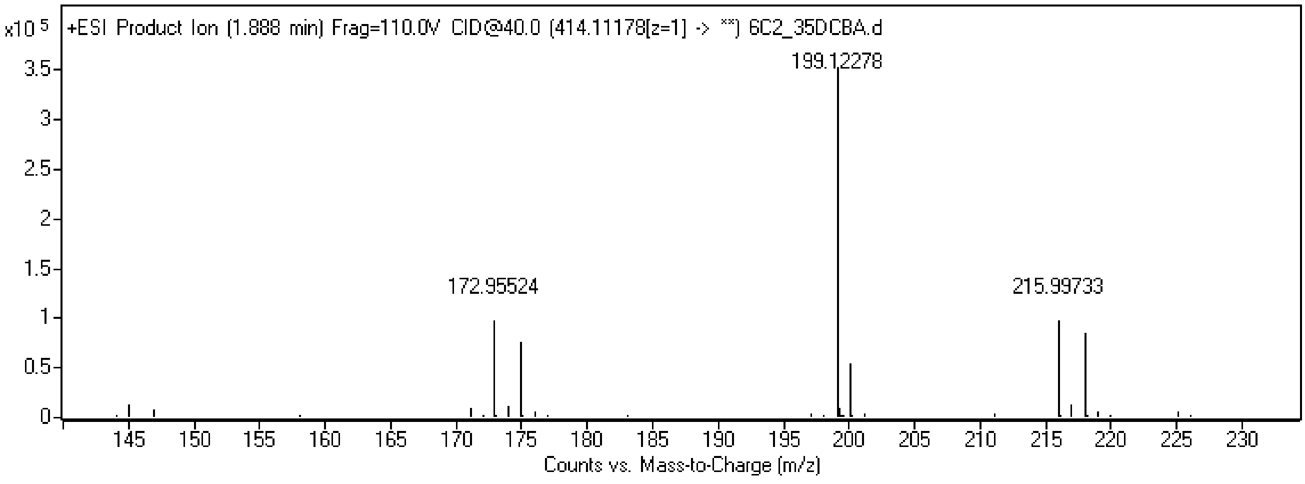

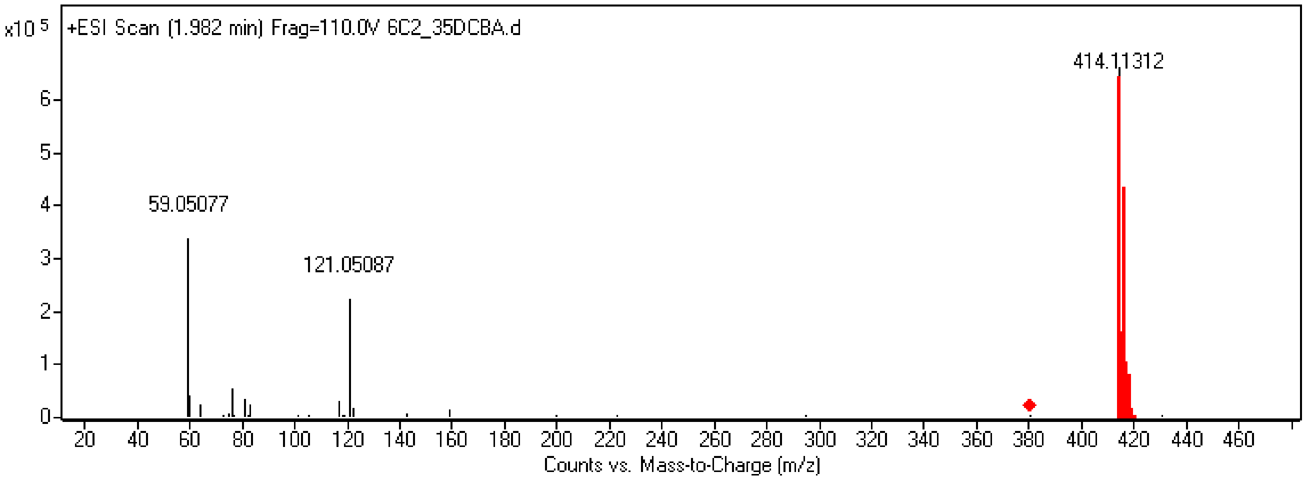

^{1}HNMR spectra for
3,5-dichloro-N-[2-(1,2,3,4-tetrahydroacridin-9-ylamino)ethyl]benzamide (2a)

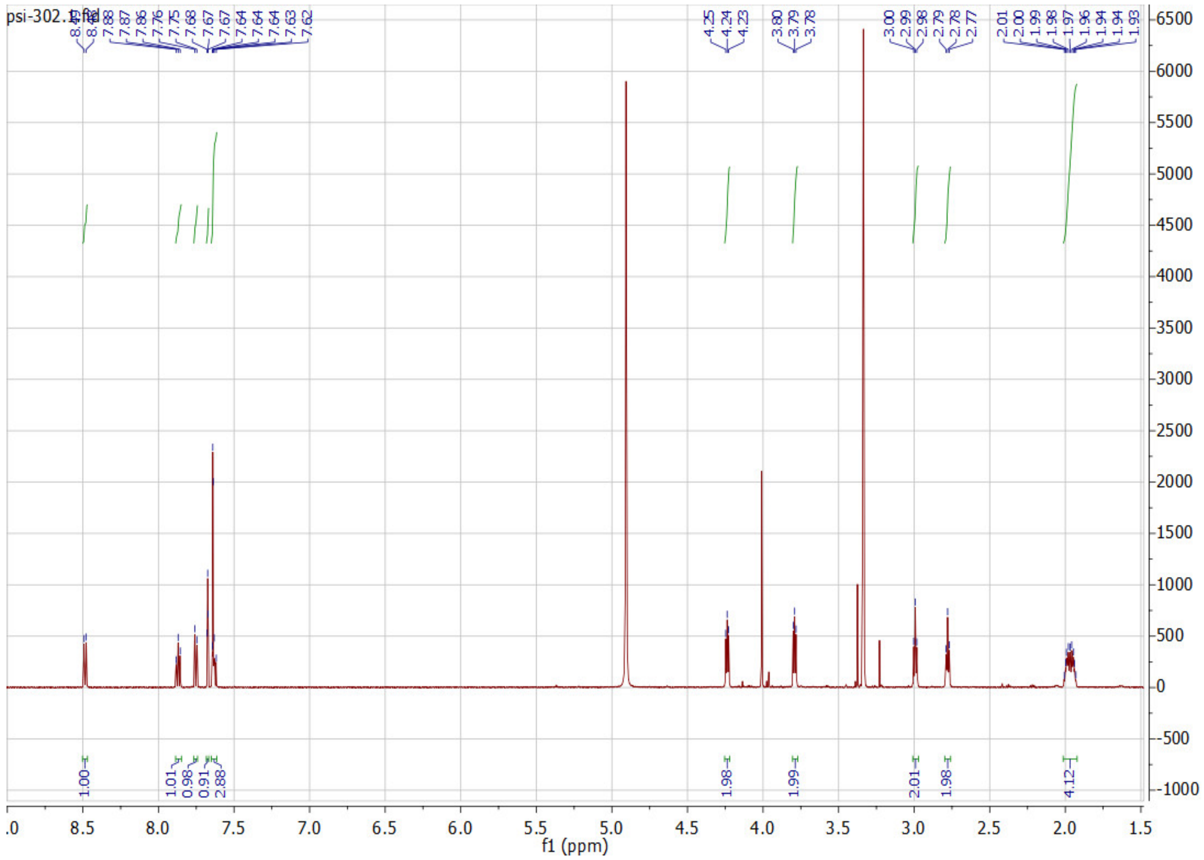

MS spectra for 3,5-dichloro-N-[2-(1,2,3,4-tetrahydroacridin-9-ylamino)propyl]benzamide (2b)

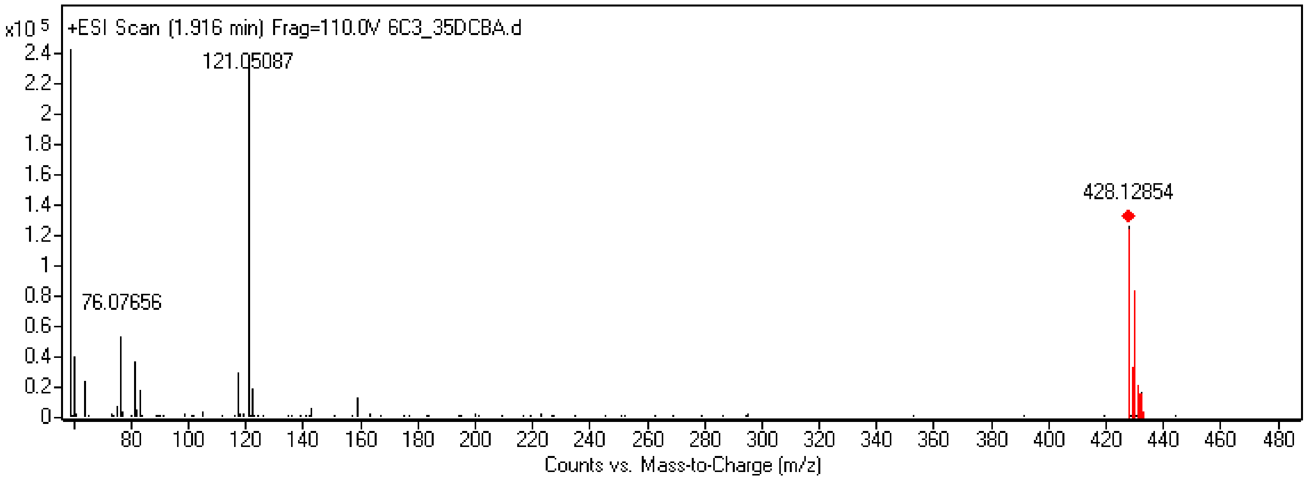

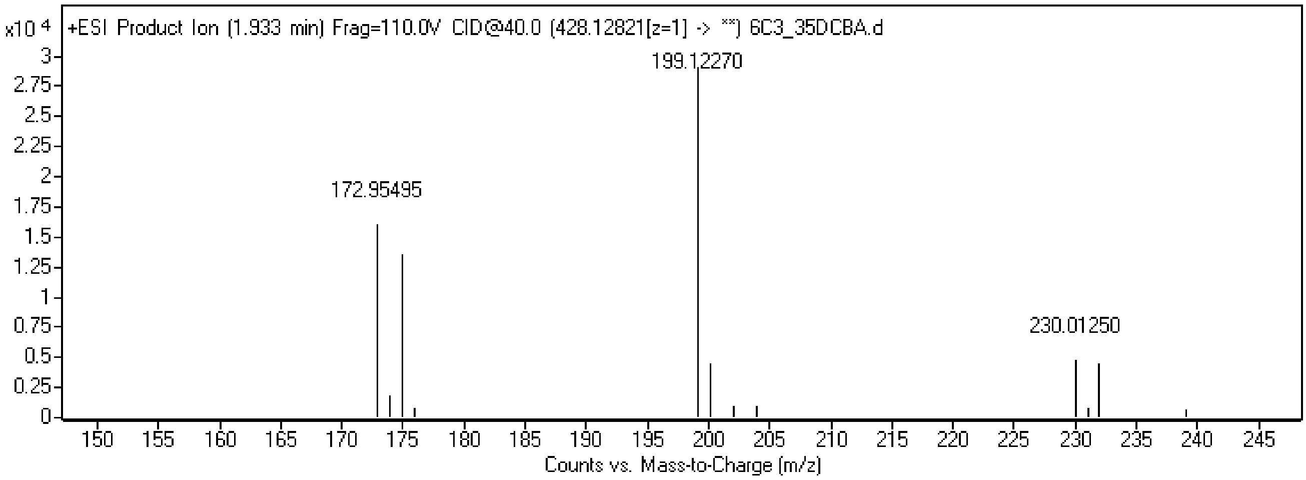

^{1}HNMR spectra for
3,5-dichloro-N-[2-(1,2,3,4-tetrahydroacridin-9-ylamino)propyl]benzamide (2b)

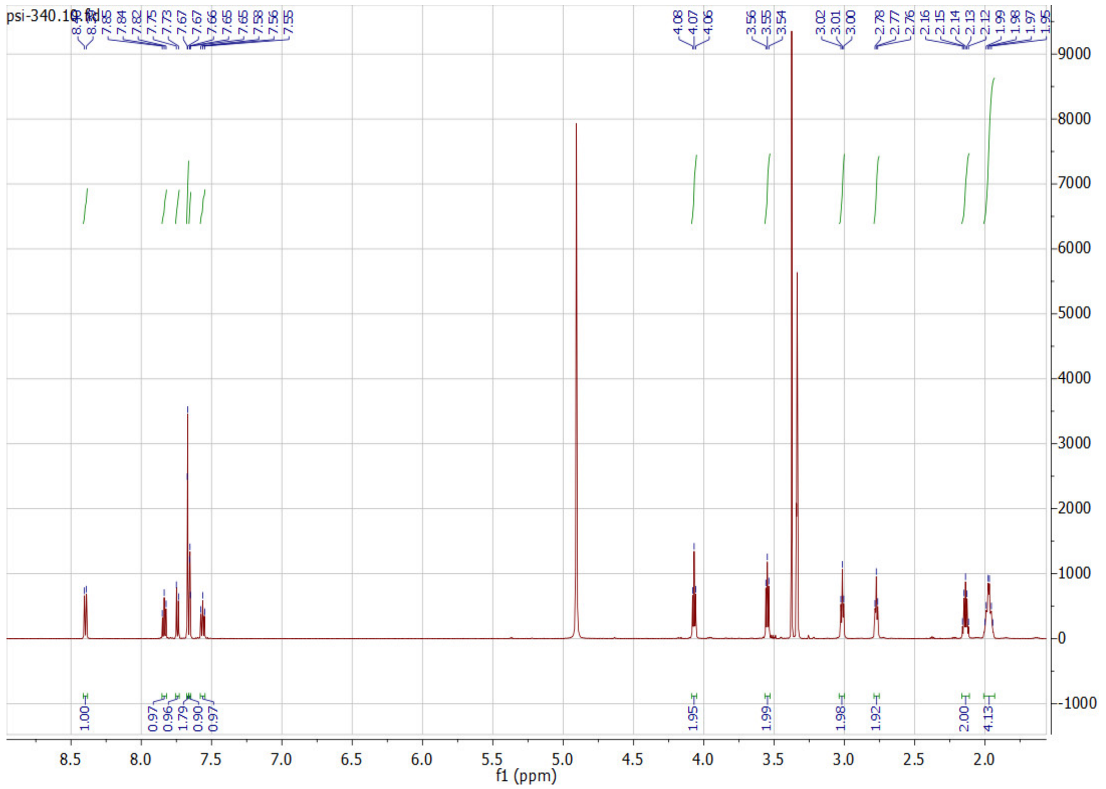

MS spectra for 3,5-dichloro-N-[2-(1,2,3,4-tetrahydroacridin-9-ylamino)butyl]benzamide (2c)

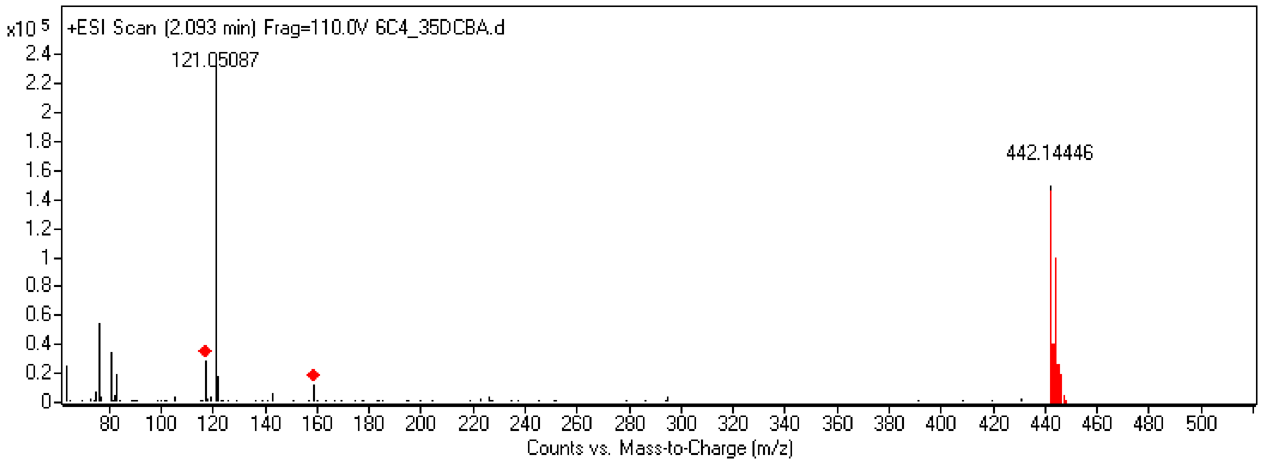

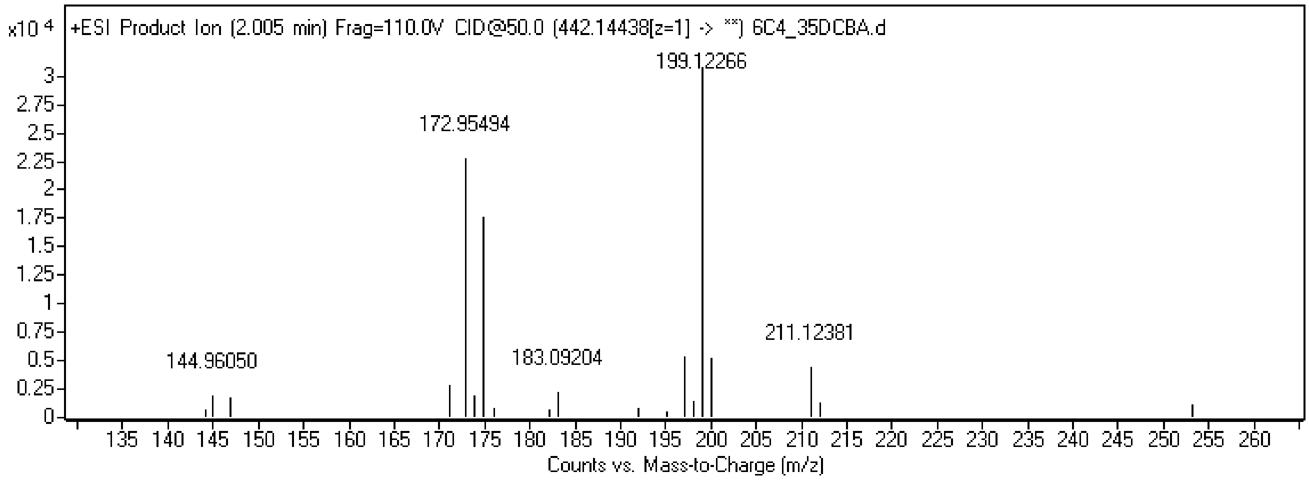

^{1}HNMR spectra for
3,5-dichloro-N-[2-(1,2,3,4-tetrahydroacridin-9-ylamino)butyl]benzamide (2c)

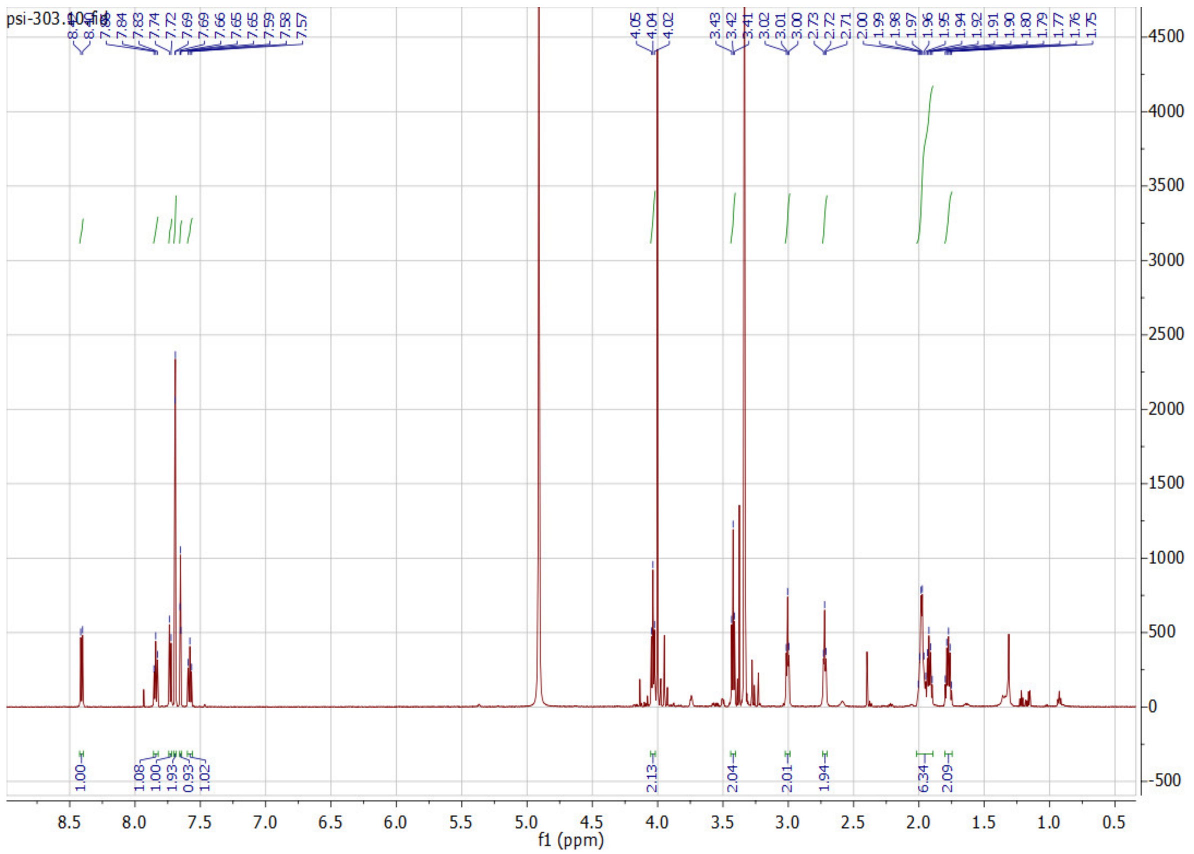

MS spectra for 3,5-dichloro-N-[2-(1,2,3,4-tetrahydroacridin-9-ylamino)pentyl]benzamide (2d)

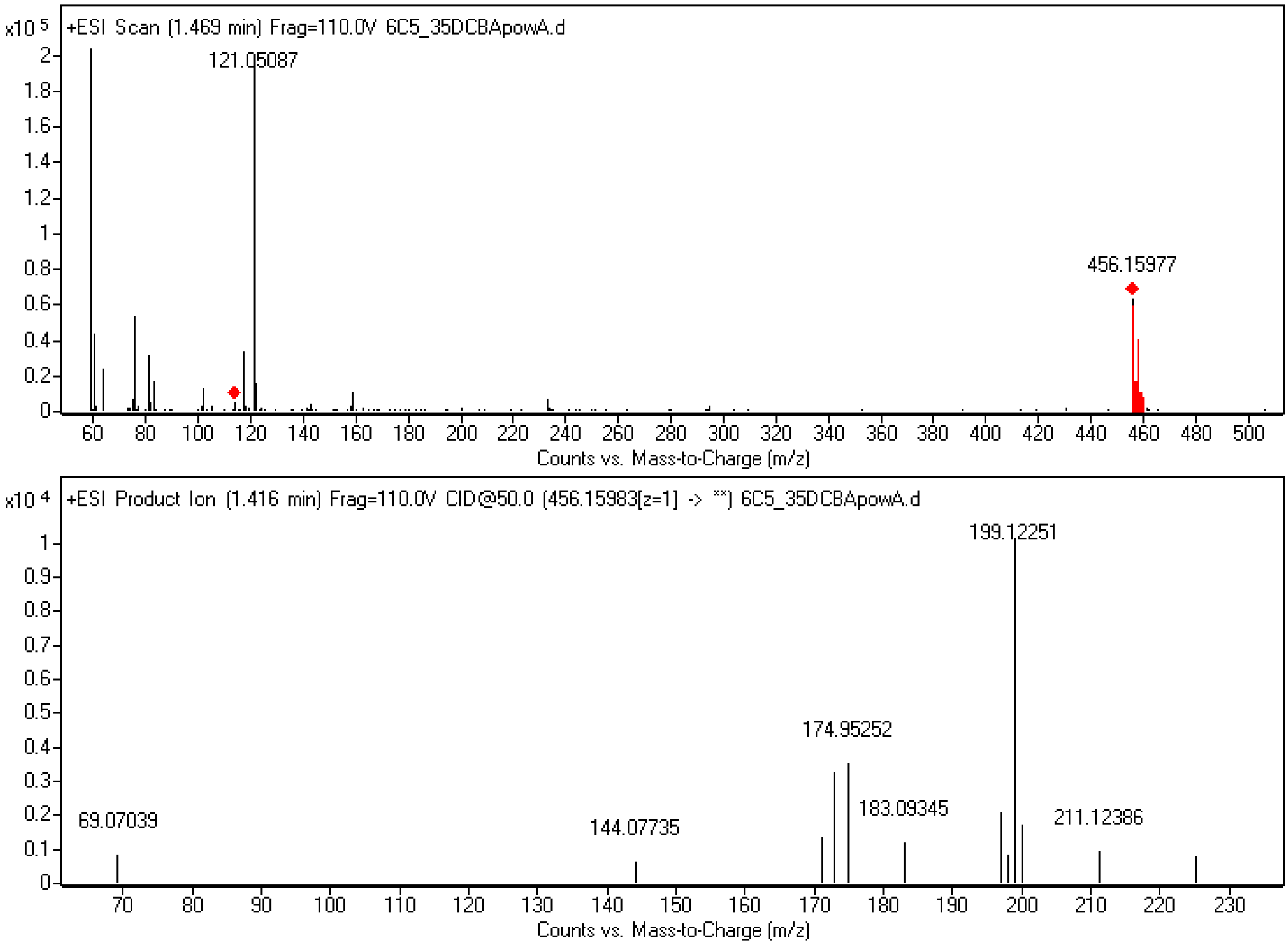

^{1}HNMR spectra for
3,5-dichloro-N-[2-(1,2,3,4-tetrahydroacridin-9-ylamino)pentyl]benzamide (2d)

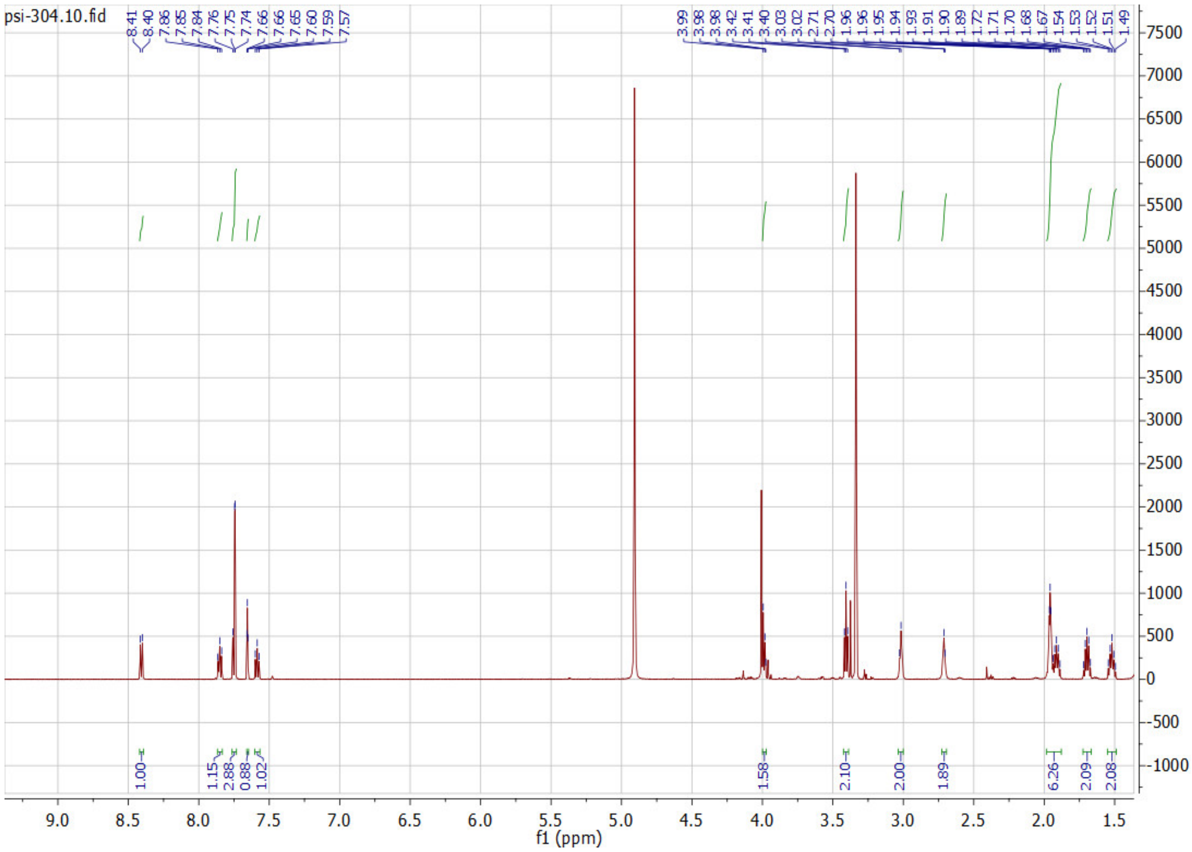

MS spectra for 3,5-dichloro-N-[2-(1,2,3,4-tetrahydroacridin-9-ylamino) hexyl]benzamide (2e)

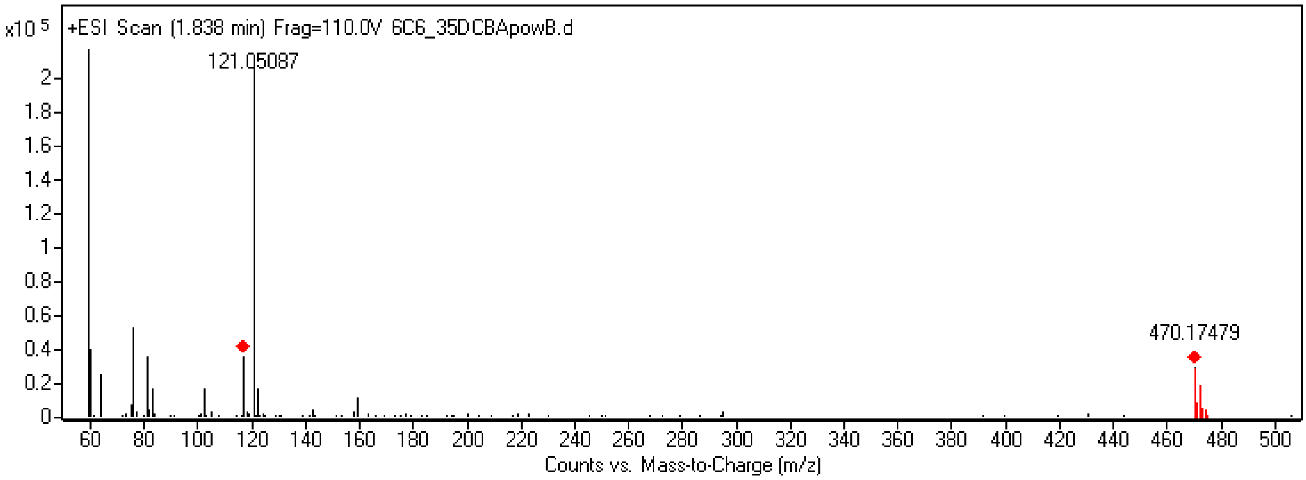

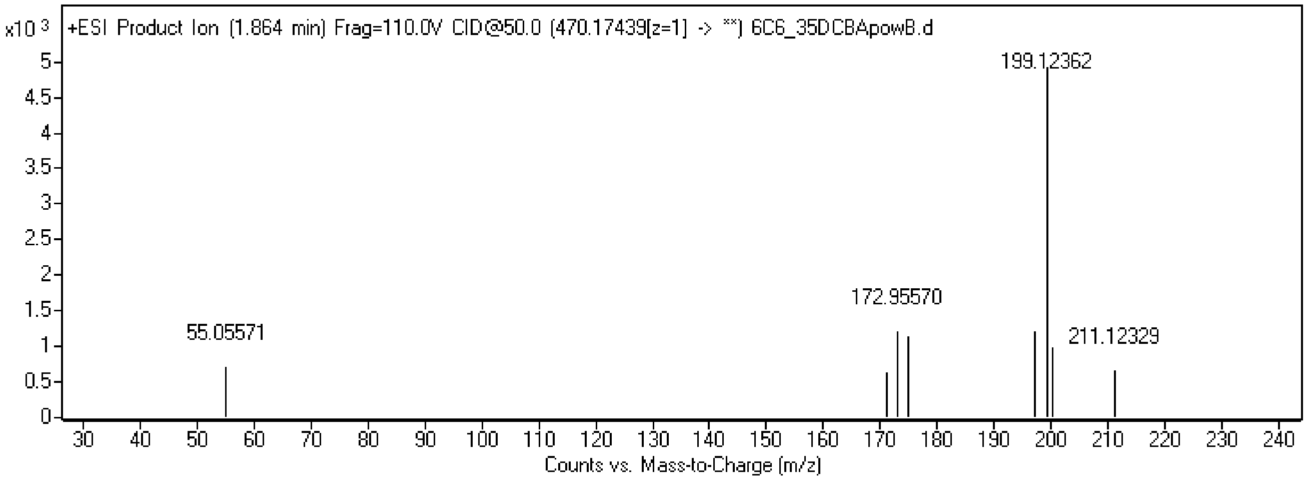

^{1}HNMR spectra for 3,5-dichloro-N-[2-(1,2,3,4-tetrahydroacridin-9-ylamino) hexyl]benzamide (2e)

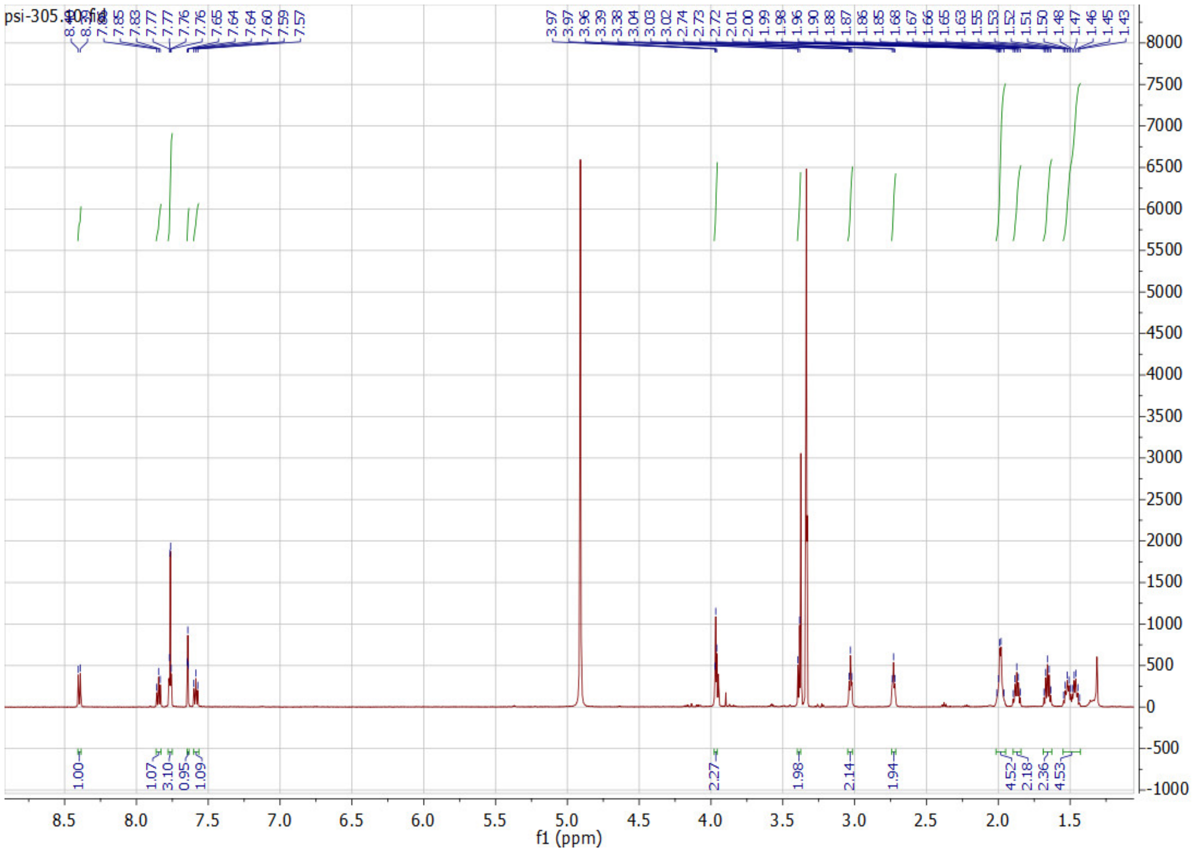

MS spectra for 3,5-dichloro-N-[2-(1,2,3,4-tetrahydroacridin-9-ylamino) heptyl]benzamide (2f)

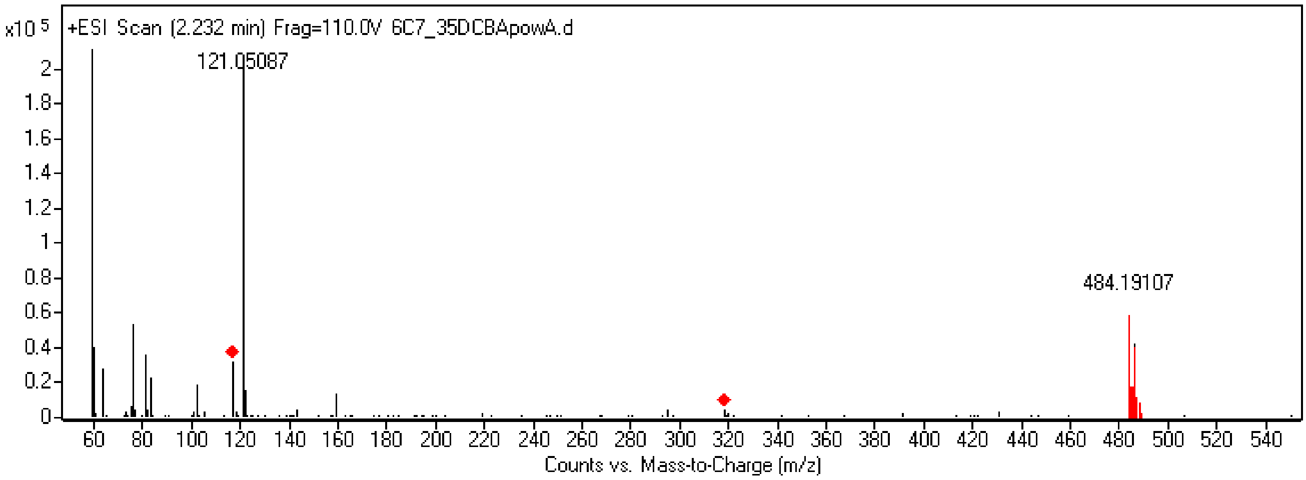

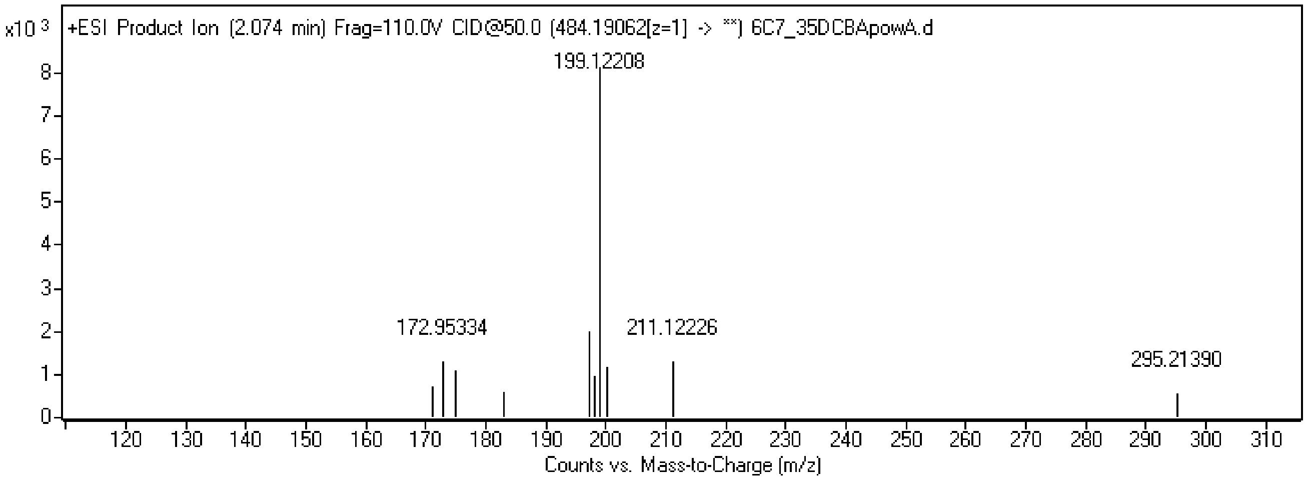

^{1}HNMR spectra for 3,5-dichloro-N-[2-(1,2,3,4-tetrahydroacridin-9-ylamino) heptyl]benzamide (2f)

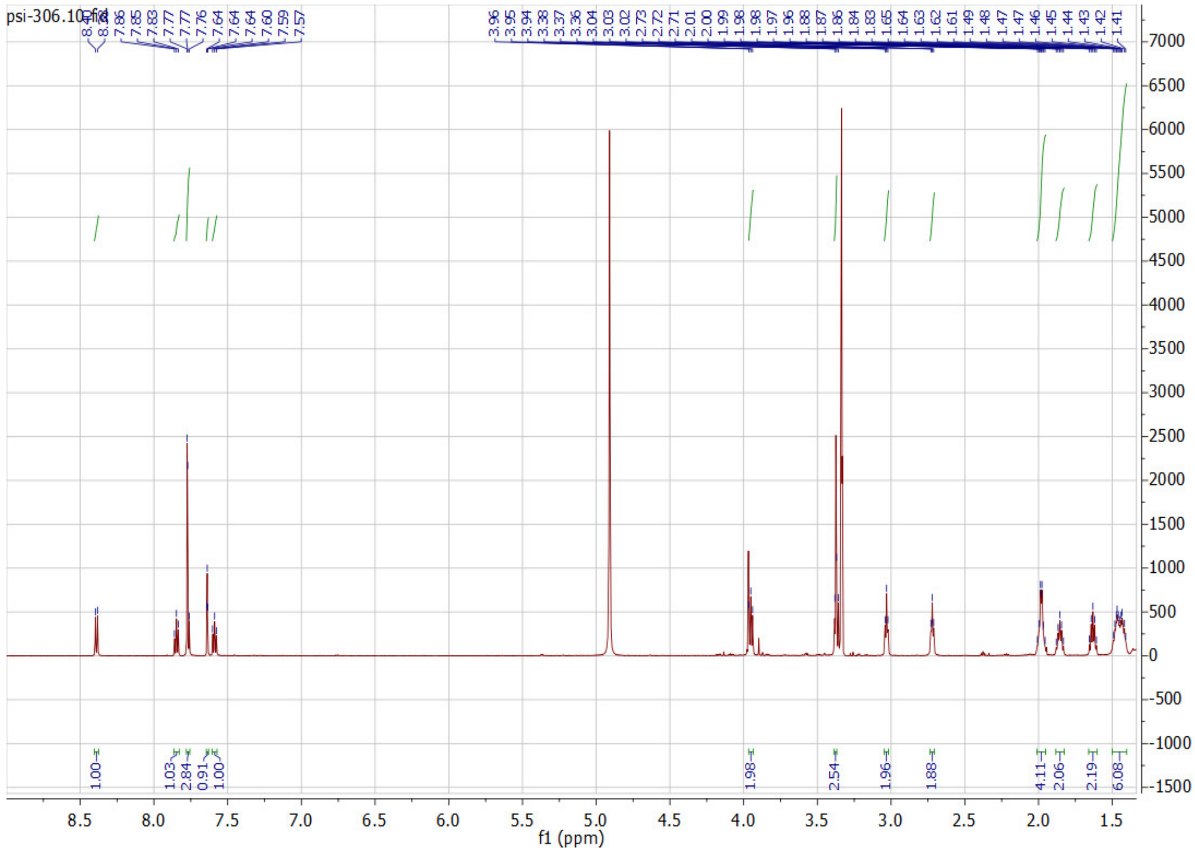

MS spectra for 3,5-dichloro-N-[2-(1,2,3,4-tetrahydroacridin-9-ylamino) octyl]benzamide (2g)

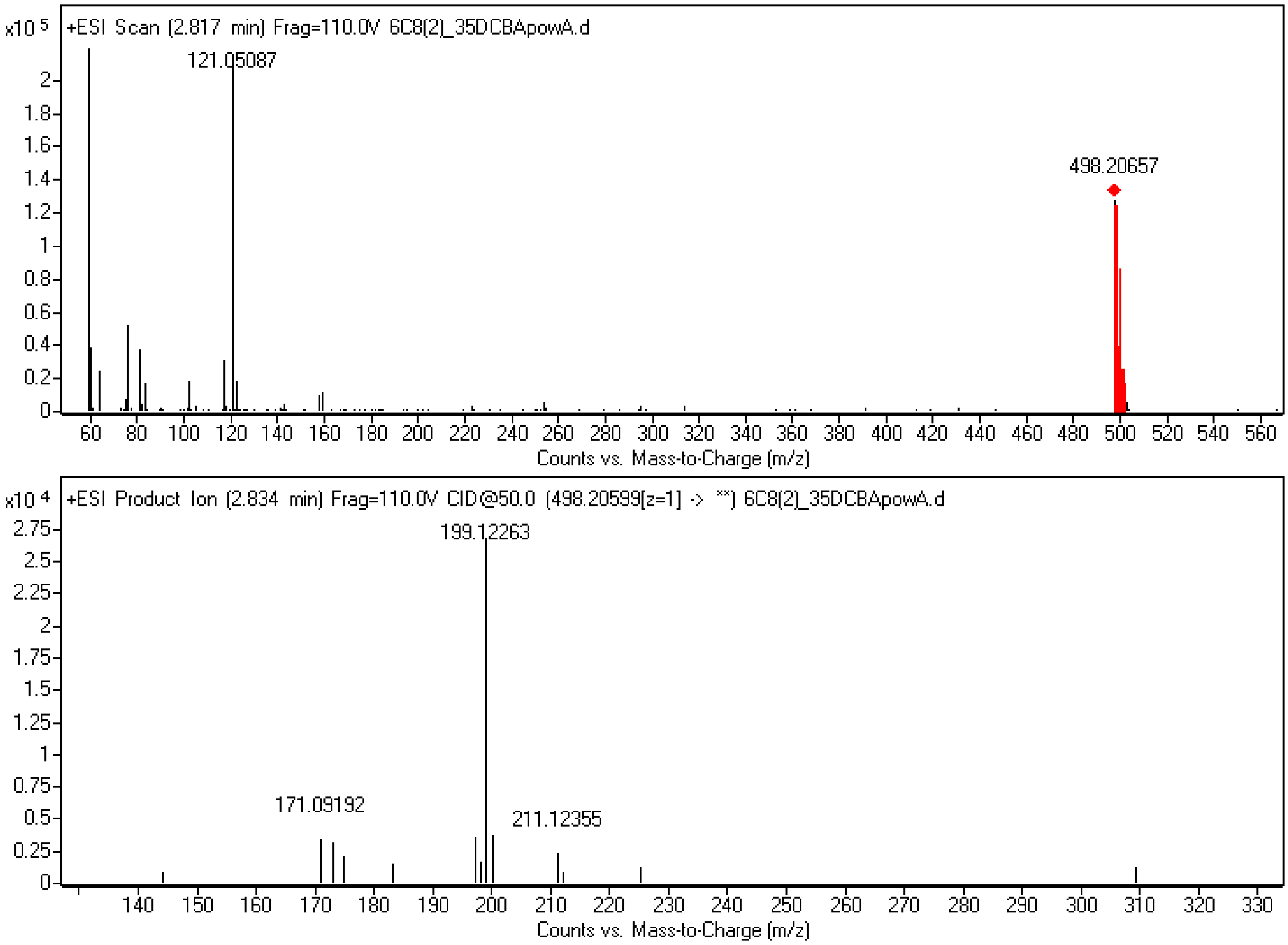

^{1}HNMR spectra for 3,5-dichloro-N-[2-(1,2,3,4-tetrahydroacridin-9-ylamino) octyl]benzamide (2g)

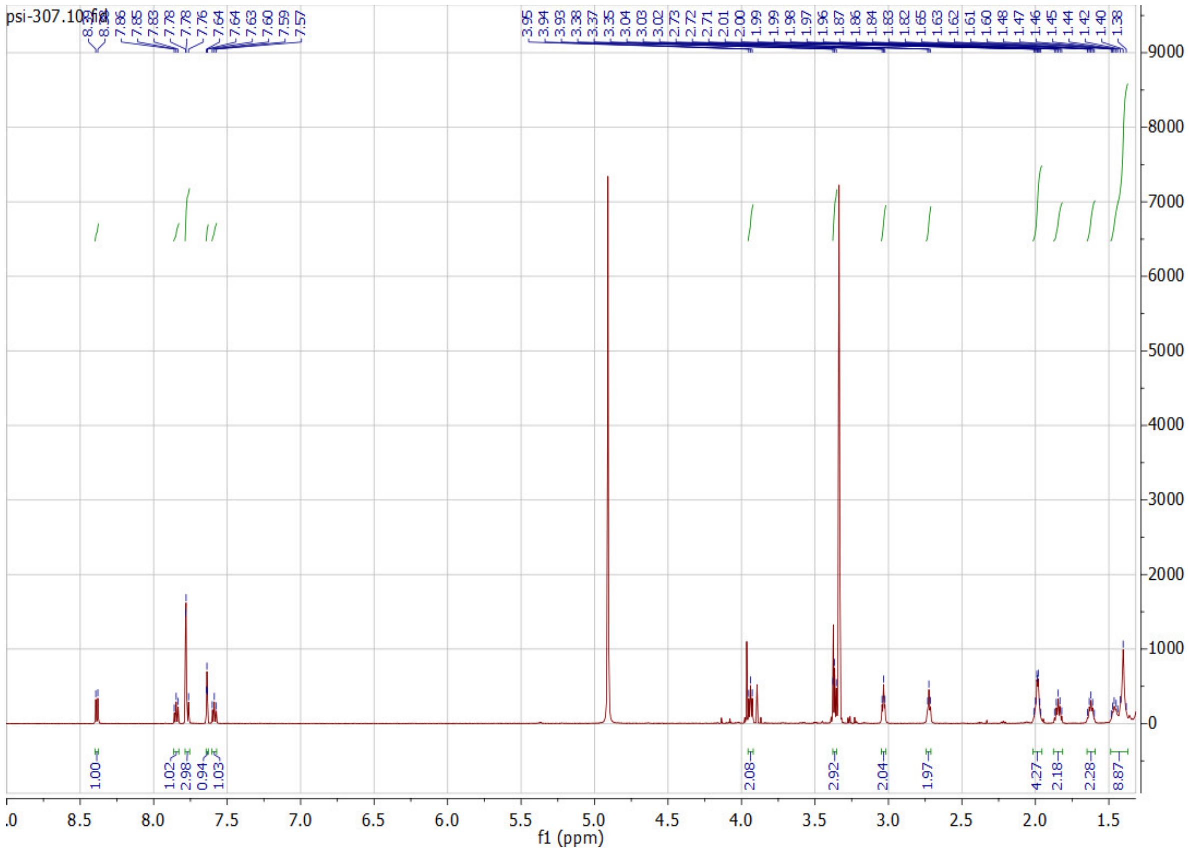

MS spectra for 3,5-dichloro-N-[2-(1,2,3,4-tetrahydroacridin-9-ylamino) nonyl]benzamide (2h)

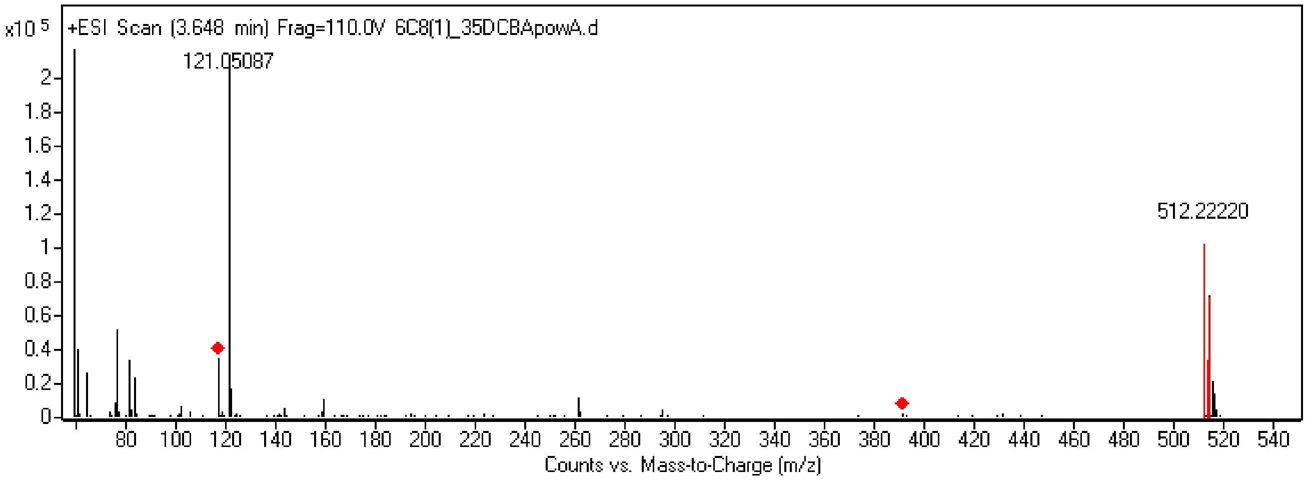

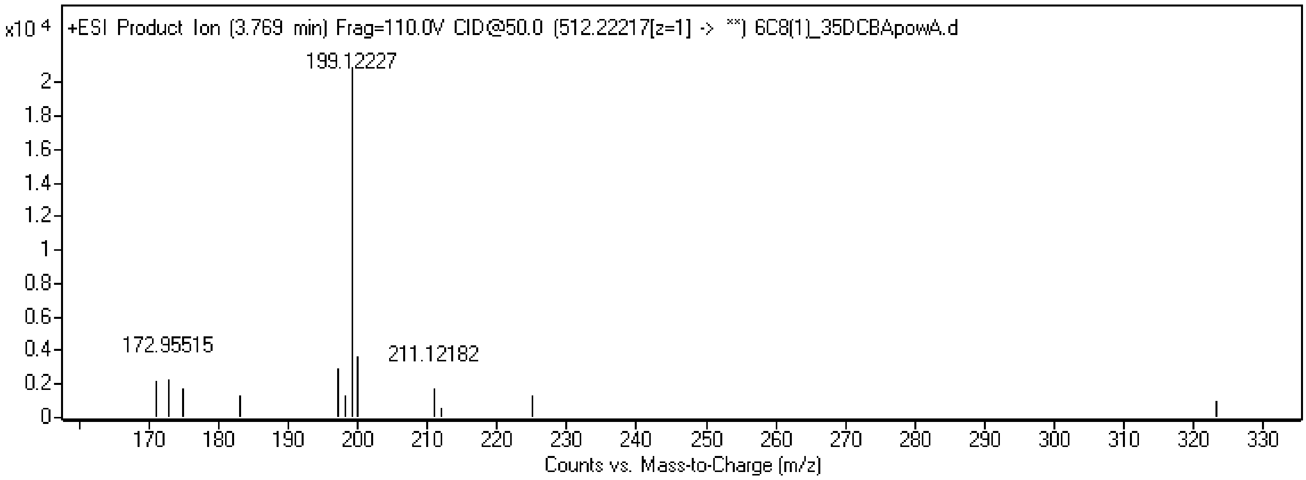

^{1}HNMR spectra for 3,5-dichloro-N-[2-(1,2,3,4-tetrahydroacridin-9-ylamino) nonyl]benzamide (2h)

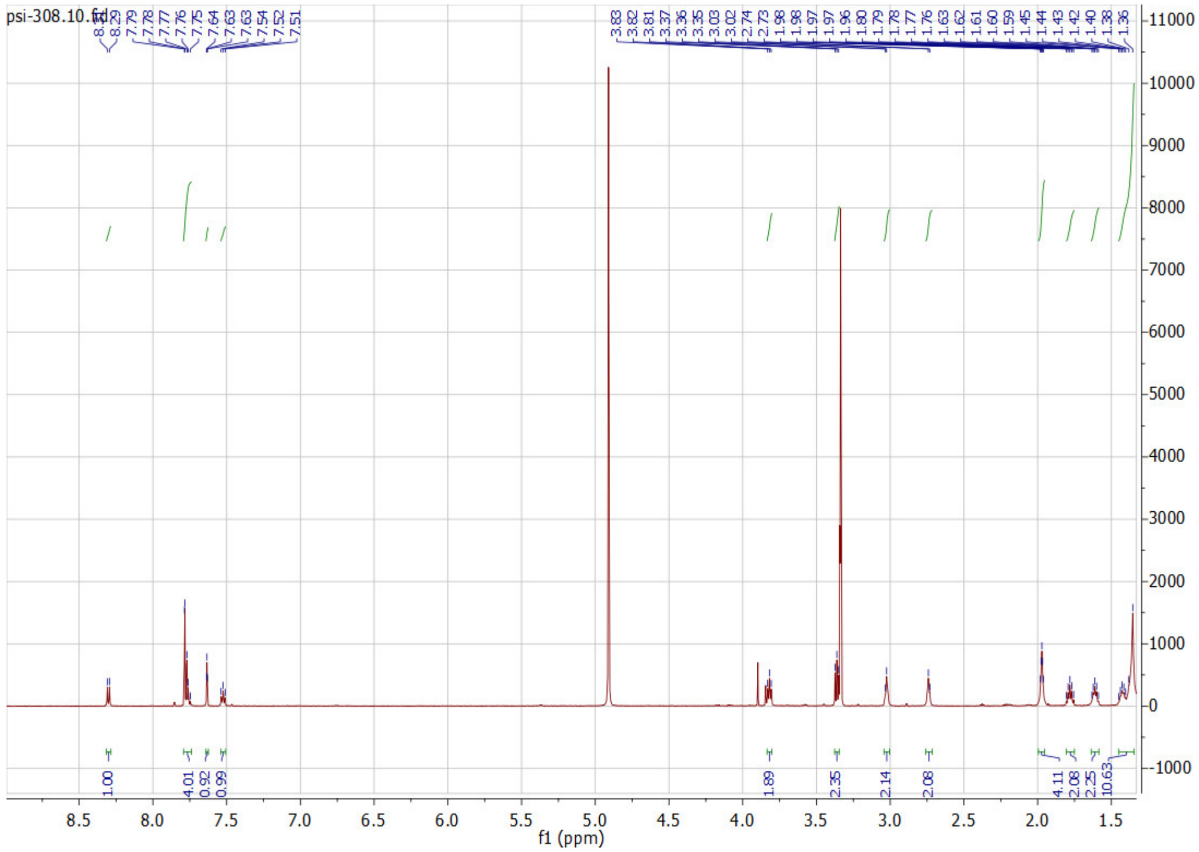

Molecular Modeling - figures with binding modes of selected compounds

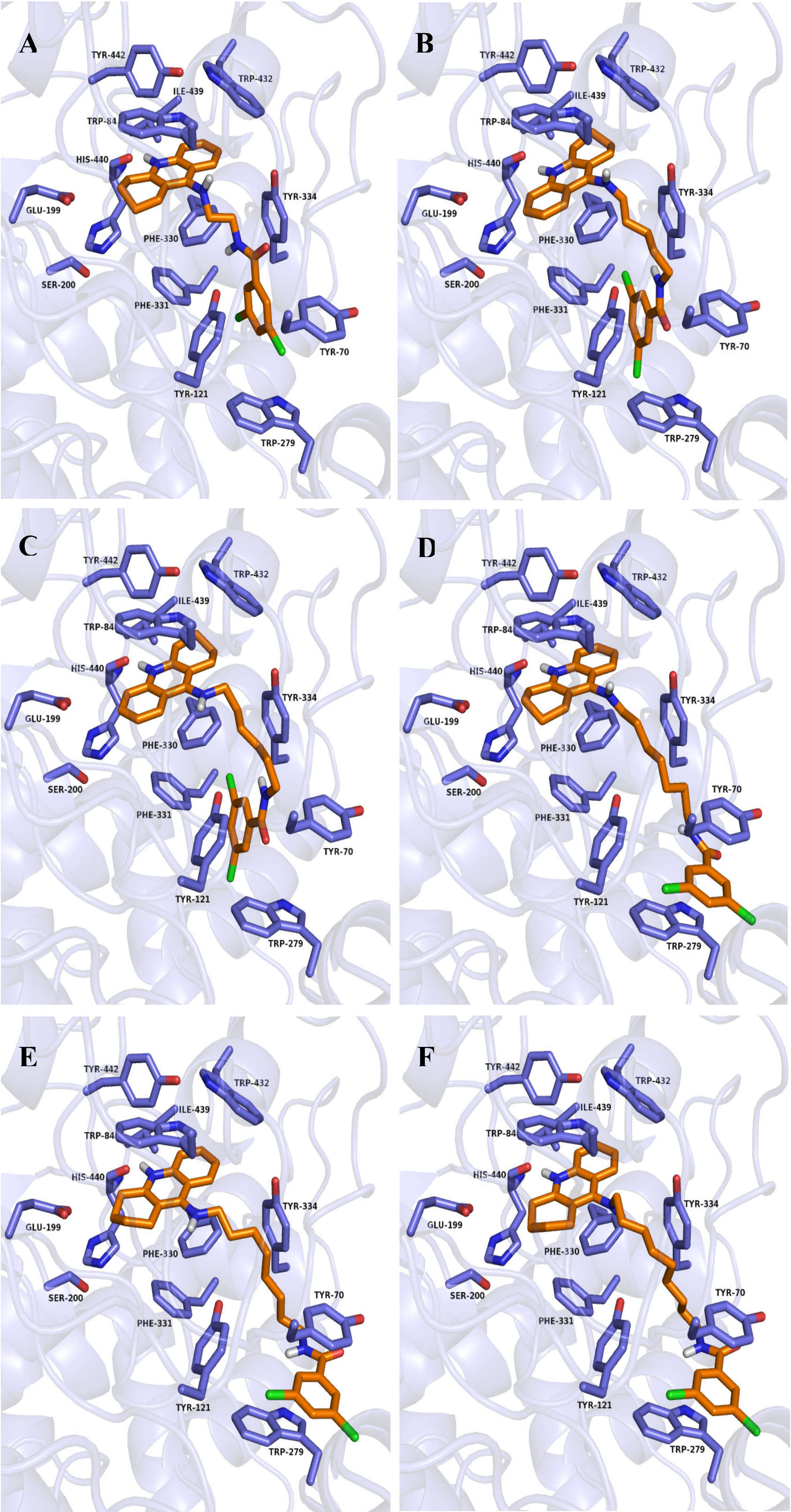

Figure 1S. Binding mode of compound **3a** **(A)**, **3d** **(B)**, **3e** **(C)**, **3f** **(D)**, **3g** **(E)** and **3h** **(F)** within acetylcholinesterase.

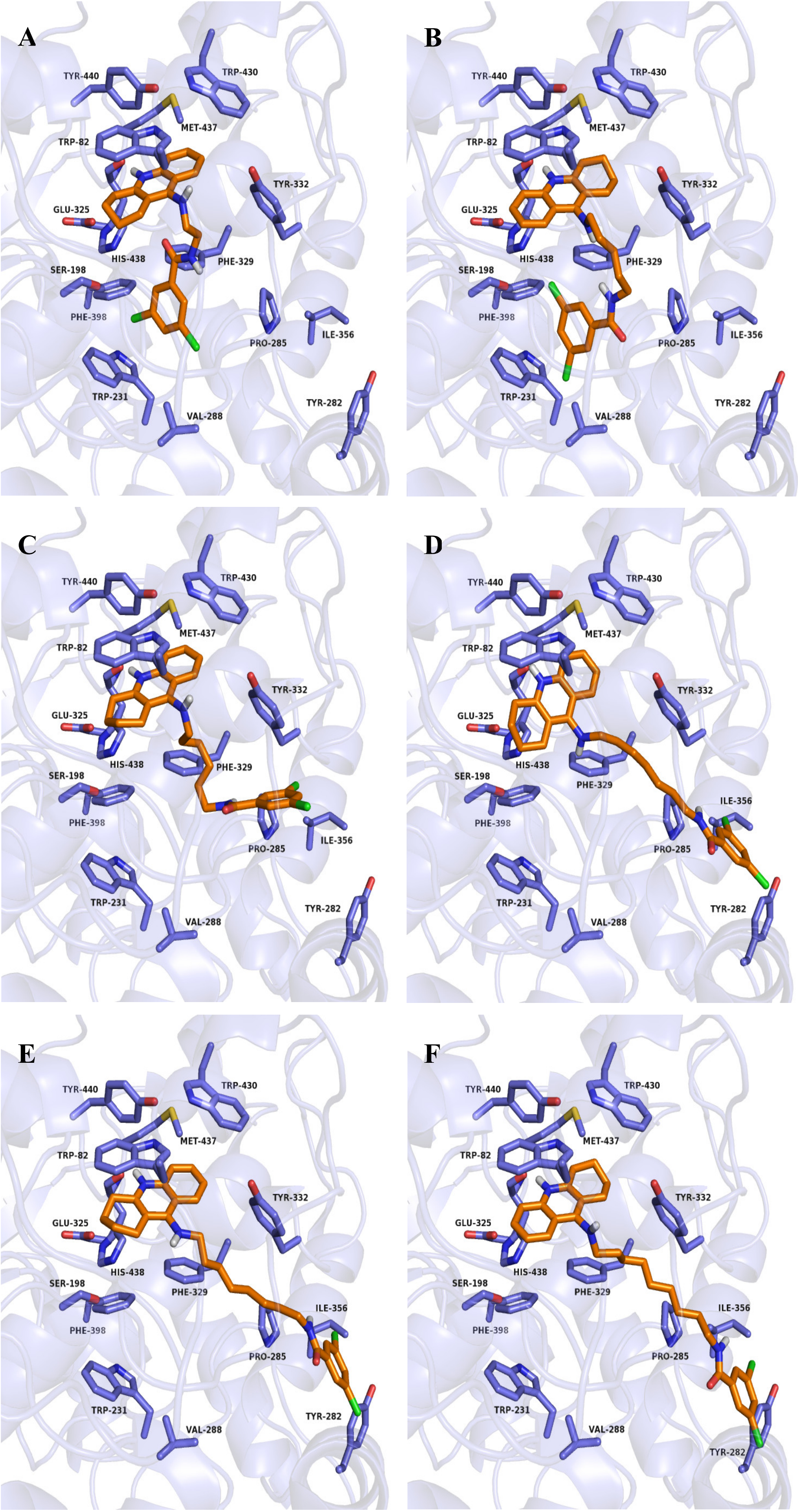

Figure 2S. Binding mode of compound **3a (A), 3d (B), 3e (C), 3f (D), 3g (E)** and **3h (F)** within butyrylcholinesterase.
